# The complete chloroplast genome sequence of *Styrax wuyuanensis* S. M. Hwang (Styracaceae) from Jiangxi Province, China

**DOI:** 10.1080/23802359.2021.1909440

**Published:** 2021-04-15

**Authors:** Rui Zhang, Yu Fan, Ying Xu, Ming Tang

**Affiliations:** College of Forestry, Jiangxi Agricultural University, Nanchang, PR China

**Keywords:** China, Styrax, Styracaceae, phylogenetic analysis

## Abstract

*Styrax wuyuanensis* S. M. Hwang is an endemic species distributed in China. In this study, we characterized its complete chloroplast genome. The circular genome of *S. wuyuanensis* is 157,969 bp in length, and includes two inverted repeat (IRa and IRb) regions of 25,954 bp in length separated by a large single copy (LSC) region of 87,575 bp and a small single copy (SSC) region of 18,486 bp. The total GC content of the *S. wuyuanensis* chloroplast genome is 37.0%, and a total of 132 functional genes are encoded, including 87 protein-coding genes, 37 tRNA, and eight rRNA. The phylogenetic analysis has shown that *S. wuyuanensis* is positioned in the Styracaceae clade, as a sister taxon to *S. faberi* and *S. fortunei*, confirming the close relationship of *S. wuyuanensis* with the latter two species.

*Styrax wuyuanensis* S. M. Hwang is a deciduous small shrub only known from the border region of Jiangxi and Anhui Provinces in China, and the type of this species is located in Wuyuan County, Jiangxi Province (Hwang 1980). This species can be easily distinguished from all other members of *Styrax* ser. *Cyrta* by its subglabrous calyx and pedicel (Li and Fritsch 2018). To date, no molecular data of the species have been reported.

In this study, we obtained and reported the complete chloroplast genome sequence of *Styrax wuyuanensis* (GenBank accession number: MW166213) based on Illumina pair-end sequencing. Fresh leaves from Wuyuan County, Jiangxi province, China (29°27′10.40″N, 117°44′16.65″E), were collected as sequencing materials. The voucher specimen (JXAU 2020026) was deposited in JXAU (the herbarium of the College of Forestry, Jiangxi Agricultural University, China. The Illumina paired-end (PE) library was prepared and sequenced in the Nanjing Novogene Biotechnology Co., Ltd. (Nanjing, China). The original reading was filtered by CLC Genomics Workbench version 9, and the clean reads were assembled by using GetOrganelle v1.5 (Jin et al. 2018). Subsequently, the plastome was annotated using GeSeq (Tillich et al. 2017) and Geneious 8.0.2 (http://www.geneious.com/), and sequences were aligned with MAFFT 7.409 (Katoh and Toh 2010).

The complete chloroplast genome of *Styrax wuyuanensis* is 157,969 bp in length, consisting of two inverted repeat regions with 25,954 bp (IRs), which were separated by a large single copy (LSC) with 87,575 bp, and small single copy (SSC) with 18,486 bp. The genome contained 132 functional genes, including 87 protein-coding, 37 tRNA, and eight rRNA. Most of the genes are single copy genes. However, seven protein-coding genes (*rpl2*, *rpl23*, *ycf2*, *ycf15*, *ndhB*, *rps7*, and *rps12*), seven tRNA (*trnI-CAU*, *trnL-CAA*, *trnV-GAC*, *trnI-GAU*, *trnA-UGC*, *trnR-ACG*, and *trnN-GUU*), and four rRNA (*rrn16*, *rrn23*, *rrn4.5*, and *rrn5*) occur in duplicate. The overall GC content of the *S. wuyuanensis* chloroplast genome is 37.0%, with the corresponding values of LSC, SSC, and IR regions as 34.8%, 30.2%, and 43.0%, respectively.

To evaluate the phylogenetic position of *S. wuyuanensis*, 23 Styracaceae chloroplast genomes together with three outgroup taxa in Symplocaceae, Ebenaceae, and Theaceae, separately, were downloaded from GenBank and analyzed. All sequences were aligned with MAFFT 7.409. The maximum-likelihood tree was constructed using RAxML 8.2.8 (Stamatakis 2014), using the GTR + Gamma substitution model and was based on 100 bootstrap replicates. *Styrax wuyuanensis* was positioned in a moderately supported clade (75%) with *S. faberi* and *S. fortunei* (Figure 1). The molecular data confirm the morpho-anatomical evidence supporting the close relationship of *S. wuyuanensis* with the latter two species, both of which have the same fundamental morphological characters as *S. wuyuanensis*, i.e. the deciduous small shrub or small tree habit, the small (0.5–1.5 mm in diameter) and rounded fruit, and so on, in *Styrax* ser. *Cyrta* (Li and Fritsch 2018).

**Figure 1.**
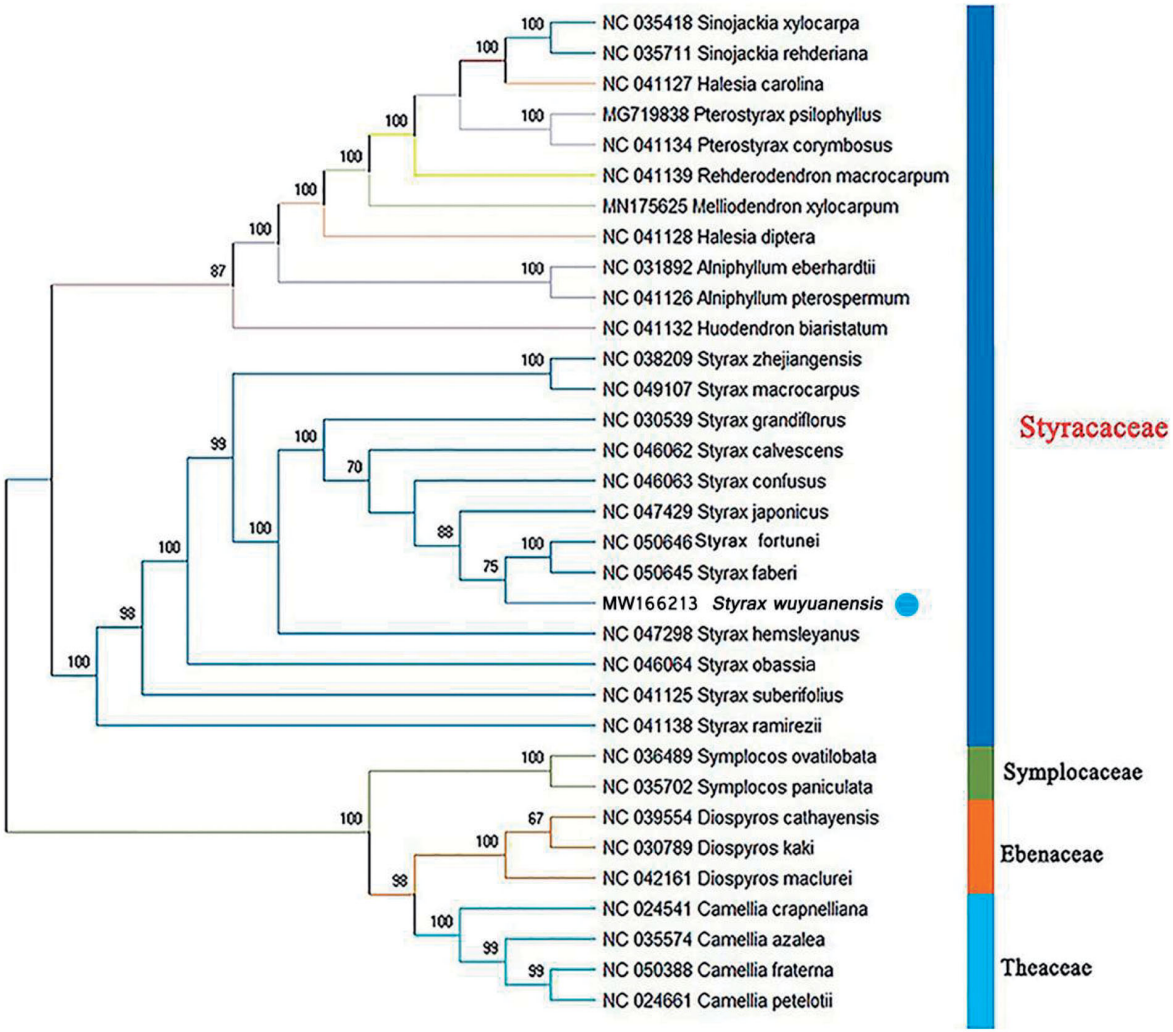
Phylogenetic tree inferred by maximum-likelihood (ML) method based on the complete chloroplast genome of 33 representative species. The bootstrap support values based on 100 replicates are shown at the branches.

## Data Availability

The data that support the findings of this study are openly available in GenBank of NCBI at https://www.ncbi.nlm.nih.gov/ under the accession no. MW166213. The associated BioProject, SRA, and Bio-Sample numbers are PRJNA692531, SRR13447960, and SAMN17348586, respectively.
